# Development of the Whole Day Matters Toolkit for Primary Care: a consensus-building study to mobilize national public health guidelines in practice

**DOI:** 10.24095/hpcdp.45.1.01

**Published:** 2025-01

**Authors:** Tamara L. Morgan, Michelle S. Fortier, Rahul Jain, Kirstin N. Lane, Kaleigh Maclaren, Taylor McFadden, Jeanette Prorok, Jill Robison, Zachary J. Weston, Jennifer R. Tomasone

**Affiliations:** 1 School of Kinesiology and Health Studies, Queen’s University, Kingston, Ontario, Canada; 2 School of Human Kinetics, University of Ottawa, Ottawa, Ontario, Canada; 3 Temerty Faculty of Medicine, University of Toronto, Toronto, Ontario, Canada; 4 Canadian Society for Exercise Physiology, Ottawa, Ontario, Canada; 5 School of Exercise Science, Physical and Health Education, University of Victoria, Victoria, British Columbia, Canada; 6 Independent Communication Specialist, Ottawa, Ontario, Canada; 7 Canadian Medical Association, Ottawa, Ontario, Canada; 8 Canadian Frailty Network, Kingston, Ontario, Canada; 9 Primary Health Care and Chronic Disease Management Network, Nova Scotia Health, Halifax, Nova Scotia, Canada

**Keywords:** health promotion, preventive health services, research methodology, physical activity, sedentary behaviour, sleep, movement behaviours, Delphi

## Abstract

**Introduction:**

Strategic knowledge mobilization efforts are needed to enhance uptake and use of the Canadian 24-Hour Movement Guidelines (24HMG), which describe optimal amounts of physical activity, sedentary behaviour and sleep each day for overall health. *The Whole Day Matters Toolkit for Primary Care* is an evidence-informed resource to help primary care providers (PCPs) disseminate the 24HMGs. The purpose of this study was to describe gaining consensus on toolkit components through iterative revisions to improve its utility in preparation for the September 2022 launch, and to summarize early dissemination efforts.

**Methods:**

A multidisciplinary expert working group planned three modified Delphi surveys to assess PCPs’ level of agreement with toolkit components on 7-point Likert scales with follow-up prompts for ratings of 4 or less. Consensus was defined a priori as a mean of 6 or higher out of 7 and 60% or more of PCPs selecting at least “somewhat agree.” Items on which consensus was reached were removed from subsequent surveys unless they were revised.

**Results:**

Twenty PCPs completed surveys 1 and 2; 15 completed survey 3. Consensus was reached on 5% (4/83), 17% (14/83) and 55% (38/69) of the items in surveys 1, 2 and 3, respectively. The number of qualitative comments decreased from 26 to 19 to 12, further indicating increasing consensus.

**Conclusion:**

Items on which consensus was not gained may reflect differences in provider characteristics or settings. A coproduced dissemination strategy was enacted. Toolkit reach was evaluated at launch and 4 months later.

**Highlights**

- We used a modified Delphi method in a rigorous, mixed methods approach to coproduce a toolkit for the 24-Hour Movement Guidelines.
- The toolkit suggests opportunities for primary care physicians in Canada to initiate discussions about adults’ current levels of physical activity, sedentary behaviour and sleep and ways to optimize these three behaviours through interventions.
- Most toolkit components were considered to be useful, understandable and relevant.
- Because a range of perspectives were considered, this toolkit can be used by diverse primary care professionals to promote the 24-Hour Movement Guidelines and therefore national public health.
- This modified Delphi approach can guide the dissemination and implementation of other public health guidelines.

## Introduction

Increasing chronic disease rates among adults are a prevailing global public health concern.^1^ Physical inactivity, excessive sedentary behaviour and poor sleep are risk factors for chronic disease and contribute to high health care costs.^2^ However, even small changes in a person’s physical activity, sedentary behaviour and sleep, or “movement behaviours,” can attenuate chronic disease risk.^3^ The Canadian 24-Hour Movement Guidelines (24HMGs) are national public health guidelines that promote sufficient good quality sleep, maximized physical activity and minimized sedentary behaviour each day.^4^ The 24HMGs guide innovative approaches to optimizing population-level movement behaviours and overall health in a 24-hour timeframe.^4^ Given that the 24HMGs are relatively new, they must be paired with effective knowledge mobilization efforts to enhance their uptake in practice and influence public health. Primary care providers (PCPs) are key to promoting the 24HMGs. However, barriers such as lack of time, confidence or belief in the efficacy of movement behaviour promotion may prevent PCPs from promoting movement behaviours among clients. These barriers must be strategically addressed to enable the successful promotion of the 24HMGs.^5^

As physical activity, sedentary behaviour and sleep are interdependent behaviours, changes to one offset time spent doing one or both of the other two, and discussing them together, rather than separately, may enhance the efficacy of movement behaviour promotion.^6^,^7^ For instance, engaging in physical activity may improve sleep while decreasing sedentary behaviour, so discussing with clients how to change multiple movement behaviours may not be a significant burden for PCPs in practice and could create additional avenues to improving population health outcomes.^7^

Until now, no known tool has existed for integrating discussions about the 24HMGs into PCP practice. We conducted a scoping review, which guided the development of “The Whole Day Matters Tool” mock-up (phase 1^8^), and a usability study, which informed its adaptation into a toolkit (phase 2^9^). The toolkit is informed by theory, following a modified 5 As (ask, assess, advise, agree and assist) counselling framework.^8^ Gaining the broad approval of the PCPs who would be using the toolkit in their practice prior to its implementation would help strengthen its mobilization. Therefore, the aim of this study was to gain consensus on the utility, acceptability and understandability (i.e. clarity) of the Whole Day Matters Toolkit for Primary Care among PCPs (phase 3) and summarize early toolkit dissemination. This article is based on a longer, previously published report.^10^

Table 1 describes the toolkit.

**Table 1:** Description of The Whole Day Matters Toolkit

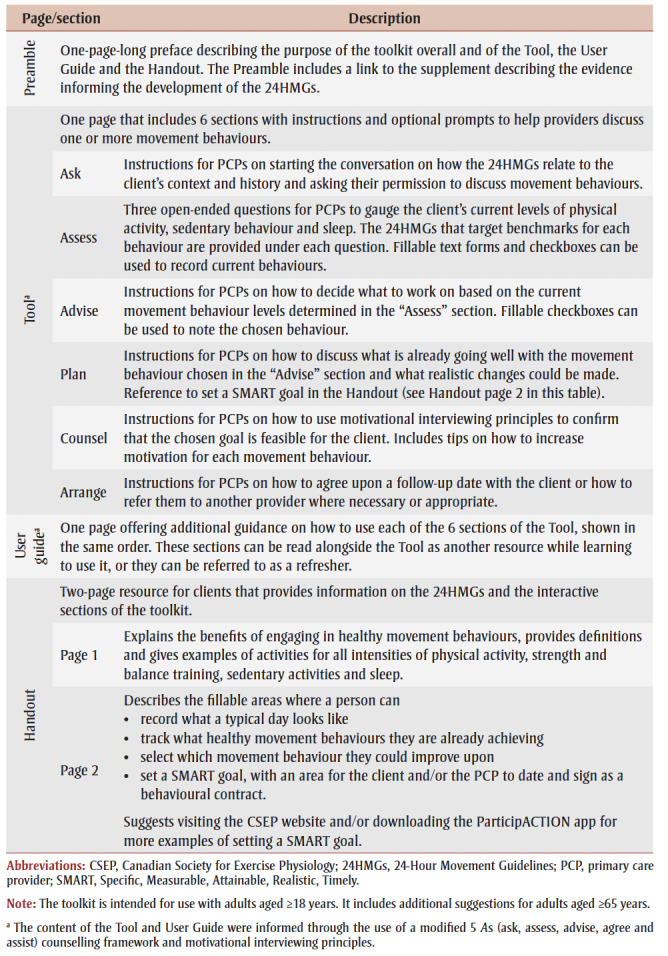

## Methods

***Ethics approval***

Institutional research ethics board approval was obtained, prior to study commencement, from the Queen’s University General Research Ethics Board (reference number: TRAQ#: 6034390).

***Guiding process***

Phases 1 to 3 of the development of the toolkit were guided by the Knowledge to Action Framework^11^ in a coproduction approach that engaged a working group of experts in public health, medicine, health promotion, kinesiology and communications throughout all the research stages.^8^,^9^,^11^,^12^ In the present study (phase 3), the working group collaborated from December 2021 to September 2022 on the ethics, study design, recruitment of participants, data interpretation and dissemination of findings.^12^ Further, critical realism supported the need for a range of ideas and processes to understand the PCPs’ evolving approval of the toolkit.^13^ We also followed the Guidance on Conducting and Reporting Delphi Studies (CREDES).^14^

***Participant recruitment and study procedure***

Feedback on the toolkit was sought from PCPs using a modified Delphi method.^15^ We modified the original Delphi method as the questions in the first survey were not entirely open-ended and we did not conduct follow-up interviews.^16^ Modified Delphi methods have commonly been used to gain consensus among health professionals, which make them the best consensus-building approach to use among geographically dispersed PCPs.^17^

This was a concurrent nested mixed methods study as we simultaneously collected quantitative and qualitative data. However, we prioritized qualitative data to achieve our study aims of gathering data to inform toolkit revisions and to guide dissemination plans.^18^ To show the diversity of perspectives across various PCP roles and in line with published recommendations,^19^ we sought to recruit five PCPs from six “categories” of eligible professions—physicians/residents, nurses/nurse practitioners, dietitians, pharmacists, psychosocial professionals (psychologists, social workers and registered psychotherapists working in a family health team), and occupational therapists working in a family health team—for a target sample of 30PCPs. A graphic and the accompanying text were posted on Twitter (now X) and Facebook inviting PCPs working in Canada to click on a link to a preliminary survey and potentially participate. Posts were also shared by the coauthors of this article to further promote the study. Twitter and Facebook were used for this convenience sampling as the coauthors’ collective professional networks spanned medical and research fields and national and local health organizations, and research shows that these two platforms are frequently used by PCPs as means of professional communication^20^ as they are quick, low-cost, broad-reaching communication tools that use existing rather than new networks across geographic and professional settings.^21^

The preliminary survey included questions to help characterize our sample, on demographics (i.e. field of work, years practising, community/ies in which the respondents work [e.g. urban, rural] and population/s they serve [e.g. adults aged 18–64 or ≥65 years, adults with chronic conditions]); self-identification (i.e. gender identity and racial or ethnic identity); knowledge and awareness of the 24HMGs (i.e. familiarity, recall of recommendations); and own 24HMG behaviours (i.e. self-reported levels of physical activity, sedentary behaviour and sleep). Eligible PCPs who provided their email address were emailed the link to the first survey (survey1). Each survey had to be completed for the respondent to be eligible for subsequent surveys. Ongoing informed consent was sought at the start of each survey. 

**Figure 1:**
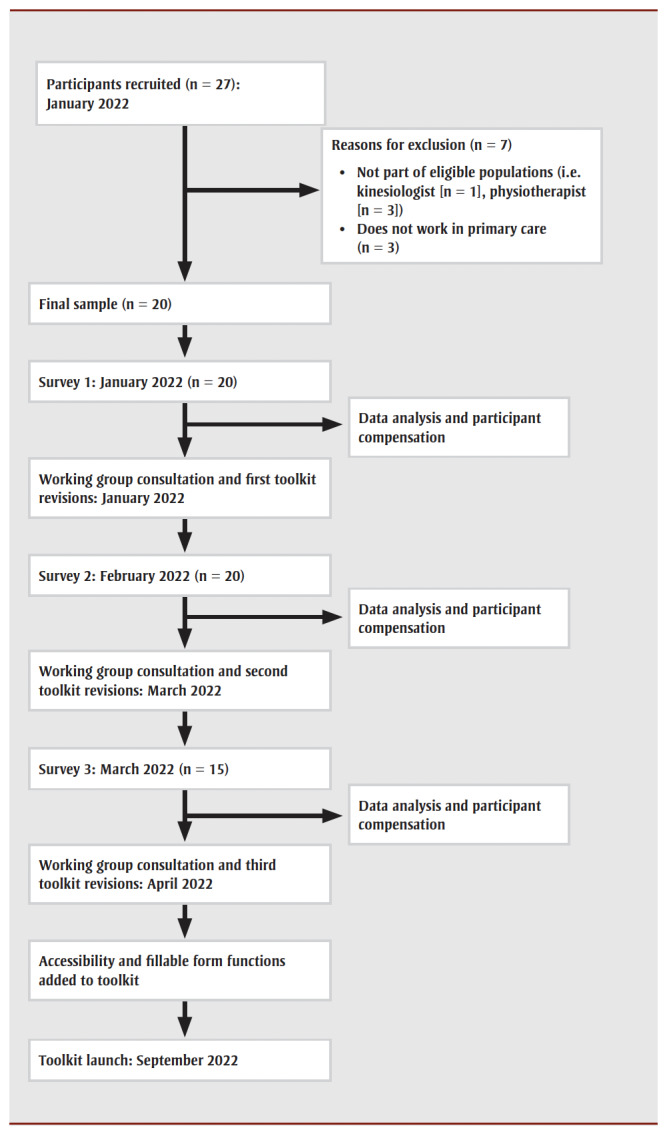
Flowchart showing the procedure for the modified Delphi surveys to revise the Whole Day Matters Toolkit for Primary Care prior to launch

Figure 1 shows the study flow procedure.

Surveys were hosted on the Qualtrics survey-making tool (Seattle, WA, US). PCPs’ agreements with survey items were recorded on a 7-point Likert scale, with 1 meaning “strongly disagree,” 4 “neither agree nor disagree” and 7 “strongly agree.” Each survey page contained questions relevant to one toolkit component (e.g. the Preamble). A text box on each page prompted participants to explain their reason for rating any items at 4 (i.e. “neither agree nor disagree”) on the Likert scale or lower and their suggested improvements, if any. Before starting surveys 2 and 3, participants were asked to review a document summarizing the results from the previous survey and the revisions made to address their comments (available from authors upon request). Up to three surveys were planned, with data collection terminated once consensus was reached on all survey items or all three surveys were completed, whichever occurred first. 

Participants were compensated with a CAD 20 e-gift card for completing surveys 1 and 2 and a CAD 35 e-gift card for completing survey 3.

***Statistical analysis***

Data from Qualtrics were exported into Microsoft Excel for Windows 10 (Microsoft Corp., Redmond, WA, US) and SPSS version 28.0 for Windows 10 (IBM, Chicago, IL, US) to calculate percentage agreement scores, mean scores, standard deviations, medians, interquartile ranges and levels of concordance. Consensus was defined a priori as a mean of 6 or greater on the 7-point Likert scale and 60% or more of participants selecting “somewhat agree” or higher per survey item (i.e. ≥5 on the 7-point Likert scale).^22^ Items for which participants did not suggest any modifications were removed from the subsequent survey and kept as-is in the toolkit. Those items that were revised based on participants’ suggestions were included in the subsequent survey to solicit further agreement on the changes. 

Dissent was defined as a lack of consensus, with more than 10% of participants presenting conflicting comments or suggesting changes to a survey item. Further, the notion of stability of ratings was applied^23^ by defining “subthreshold consensus” as items on which a mean score between “somewhat agree” and “agree” (i.e. between 5 and 6 on the 7-point Likert scale) was reached and on which either favourable comments, suggested changes from less than 10% of participants, or no suggested changes were provided. Differentiating dissent from subthreshold consensus allowed us to distinguish between those items for which there was no consensus, those for which there was qualitatively suggested disagreement (i.e. relatively lower utility, acceptability and understandability) and those for which there was agreement (i.e. relatively greater utility, acceptability and understandability). Qualitative data were used to interpret quantitative ratings and were the primary drivers of toolkit revisions. To make the revisions, participants’ suggested changes were noted following each survey analysis and discussed among the working group during 90-minute virtual meetings and via email correspondence to collaboratively decide how to best address these comments. These discussions either resulted in the toolkit item being revised (e.g. by changing the wording, design or order) or removed (i.e. if participants found it to be redundant, unhelpful or not visually appealing). 

Levels of association between participants’ ratings were analyzed for each survey using the Kendall coefficient of concordance (*W*) as the data were non-parametric and all surveys had more than two raters (i.e. participants).^24^ Chi-square (χ^2^) analyses were run to assess for statistical significance of *W* using 95% confidence intervals.

***Validation***

To externally validate the toolkit, we contacted the researchers who led the systematic reviews that informed the physical activity, sedentary behaviour and sleep recommendations of the 24HMG, who are experts in their fields in Canada, and asked for their feedback on content accuracy. 

***Dissemination***

We codeveloped and coenacted a dissemination strategy, which included creating and hosting a short video on the Canadian Society for Exercise Physiology (CSEP) 24HMGs website; posting about the toolkit on the Canadian Medical Association’s Physician Wellness Hub; e-blasting the professional networks of CSEP, the Canadian Frailty Network, the 24HMGs Knowledge Translation Advisory Committee and Guideline Development Consensus Panel, and the Health Promotion team at Queen’s University; and posting on Twitter (10 posts), Instagram (8 posts, 5 stories), Facebook (7posts, 1 story), LinkedIn (2 posts) and YouTube (2 videos). This dissemination strategy was put into practice on the day the toolkit was launched, and metrics were collected at one week and four months post launch to evaluate toolkit reach.

## Results

Twenty-seven individuals completed the intent to participate form, but seven were ineligible because they worked outside of primary care (n = 3) or were not in an eligible population (n = 4). Twenty PCPs working in British Columbia, Alberta and Ontario were eligible to participate, consented to do so and completed surveys 1 and 2. Despite email reminders, five PCPs did not complete survey 3. This sample size of 20 was lower than intended, and we were unable to extend our recruitment period; however, many Delphi studies have used 15 to 20 participants.^25^

Table 2 displays participant characteristics.

**Table 2:** The Whole Day Matters Toolkit for Primary Care Providers consensus-building study participant demographic and occupational characteristics

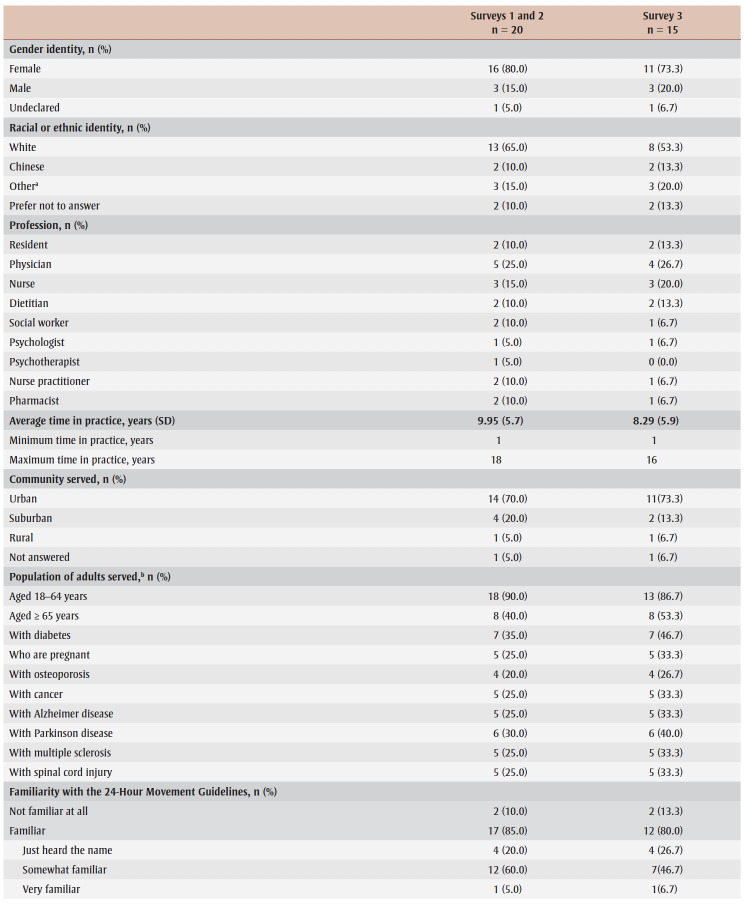 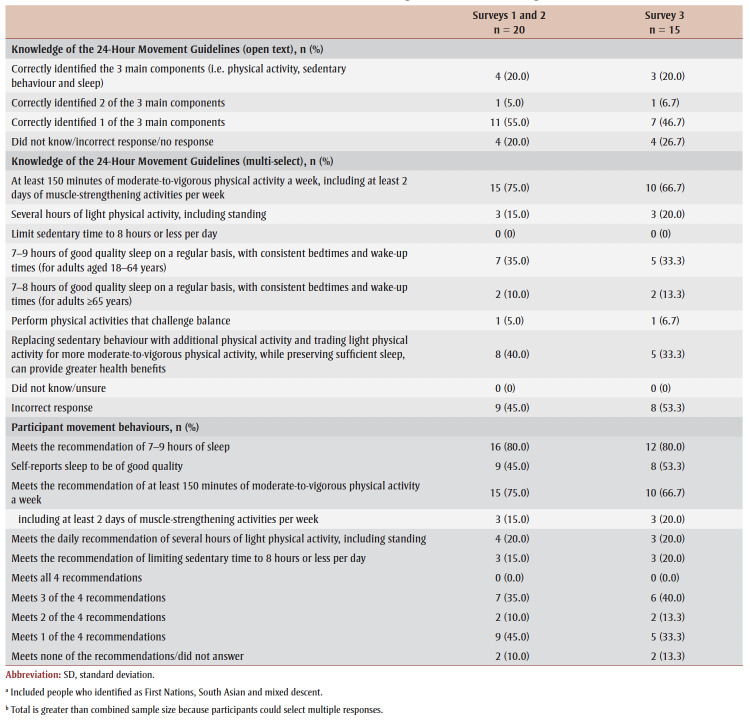

Percentage agreement scores, means, standard deviations, medians and interquartile ranges from surveys 1 to 3 are shown in Table 3. Across all surveys, the percentage agreement criterion was met 100% of the time, while the mean score criterion was met in increasing proportions with each survey. In survey 1, participants reached consensus on 5% (4/83) of the toolkit items and made 26 qualitative comments. The item “I think [adults accessing care] would use the Handout” received the lowest mean score of 5.4 out of 7. The participants reached consensus on the following items: that they would want to use the “Advise” and “Arrange” sections and the User Guide in their practice; and that adult clients would understand the Handout. Qualitative comments were about how the Preamble, Tool, User Guide and Handout were too busy and text-heavy (e.g. “It is a lot of text but I understand the need to provide this information for accurate use of this Tool.” [P01]). 

**Table 3:** Results from surveys 1 to 3 of the Whole Day Matters Toolkit for Primary Care Providers consensus-building study

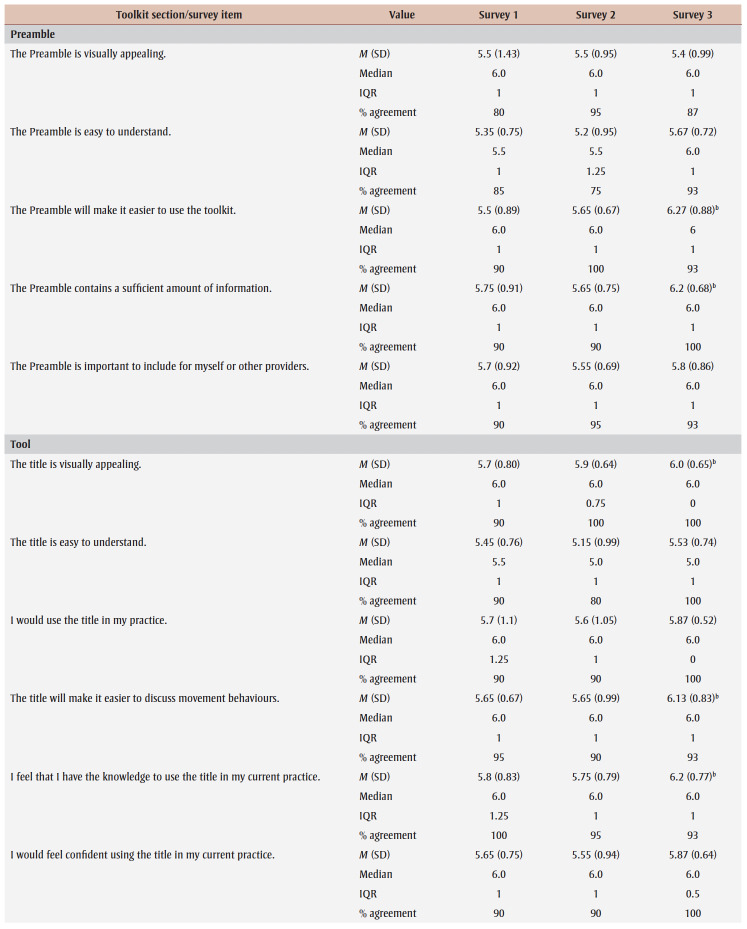 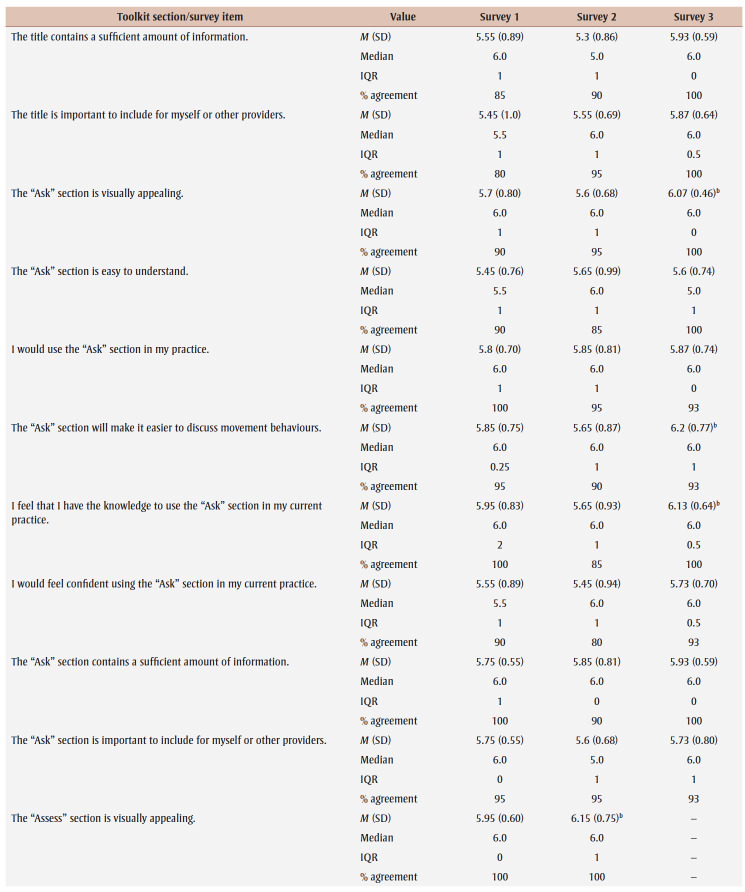 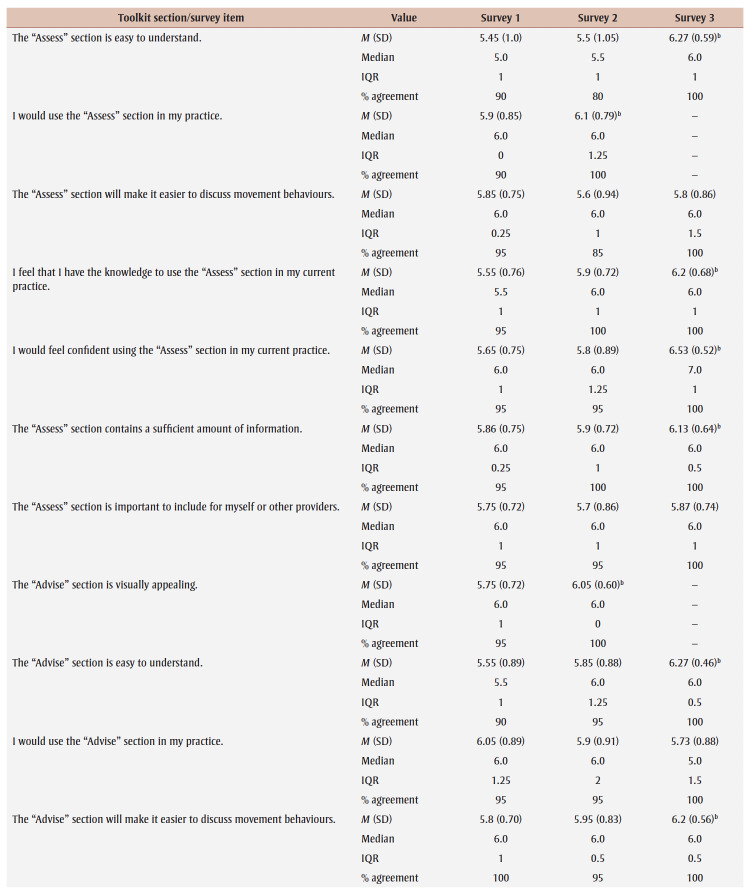 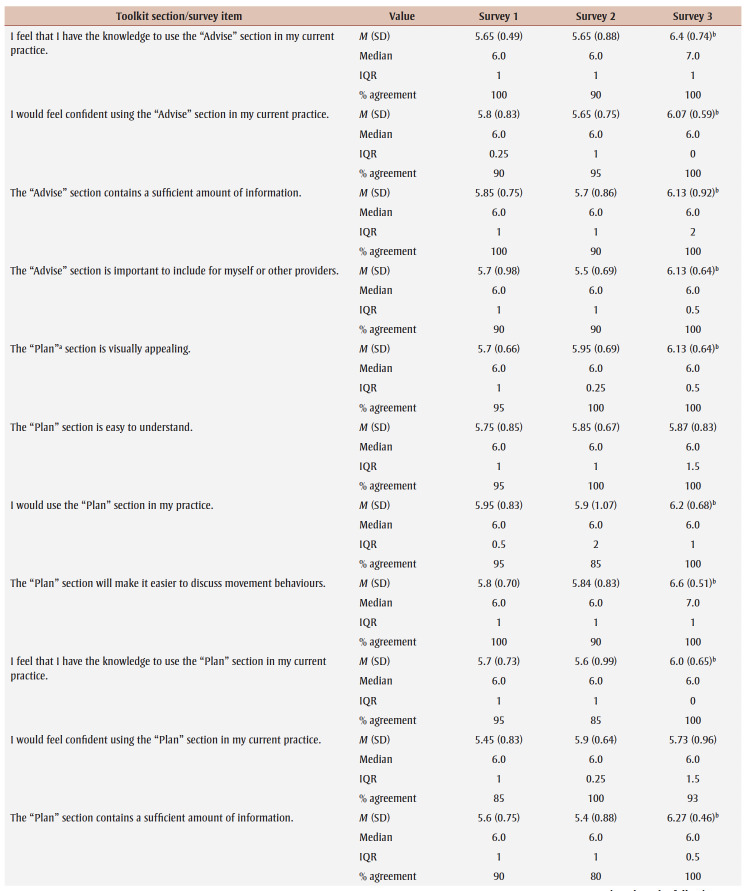 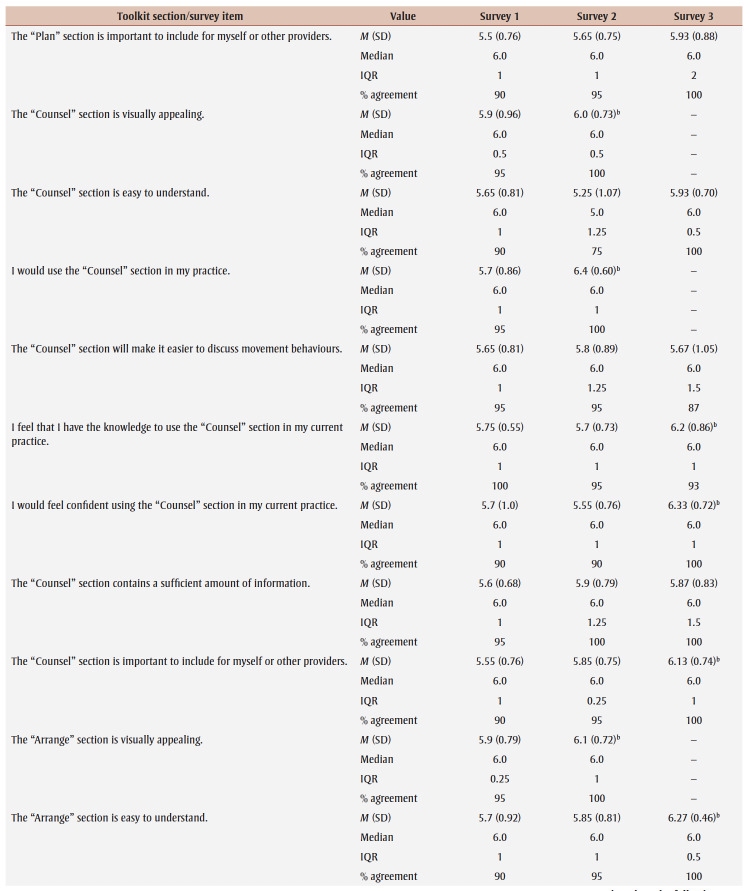 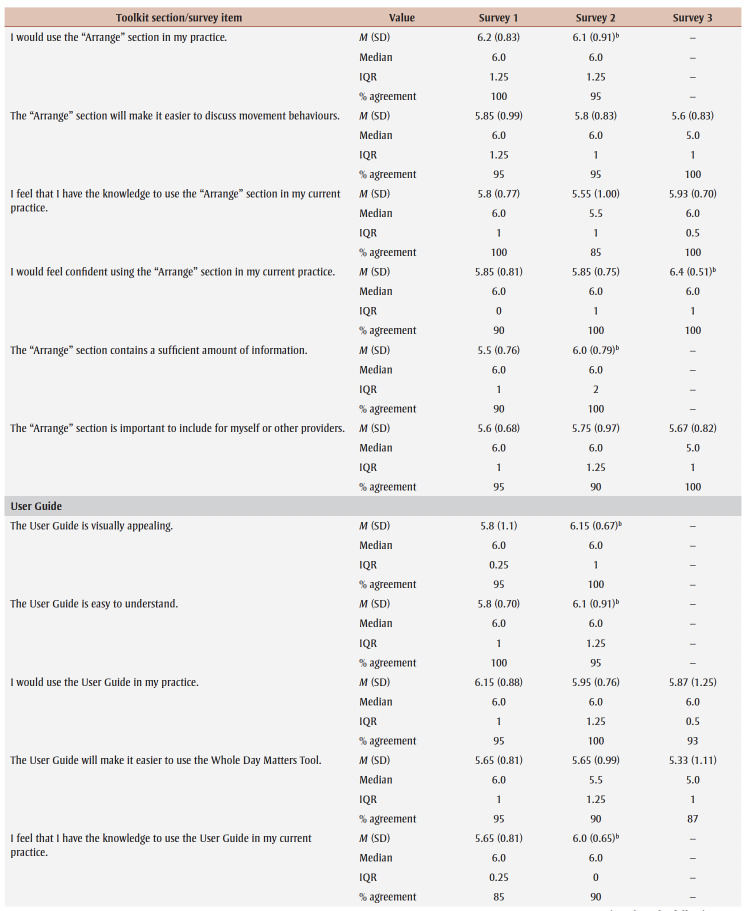 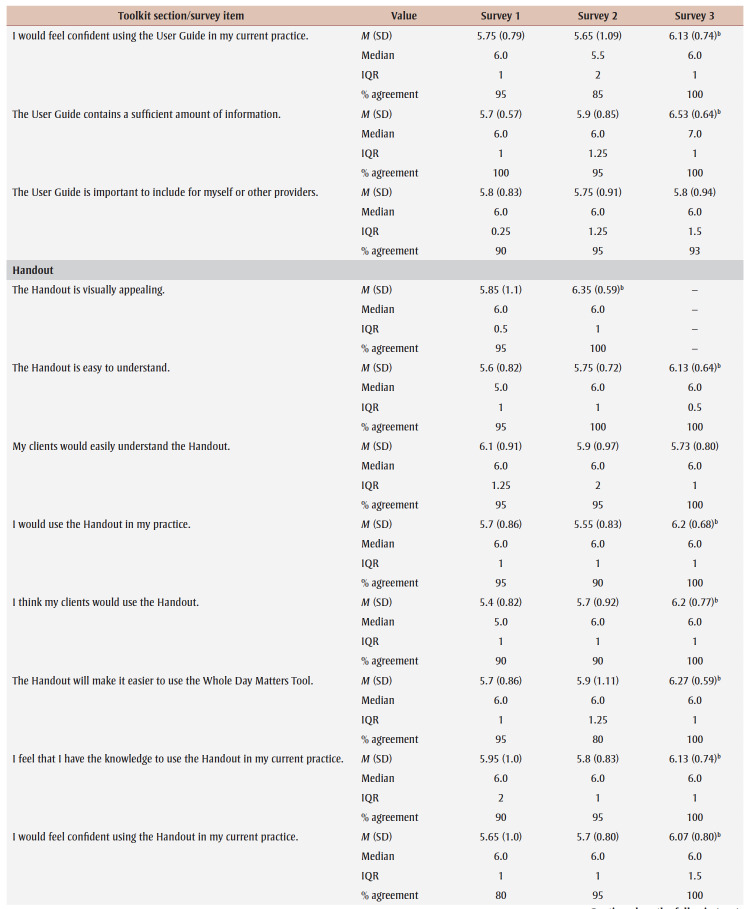 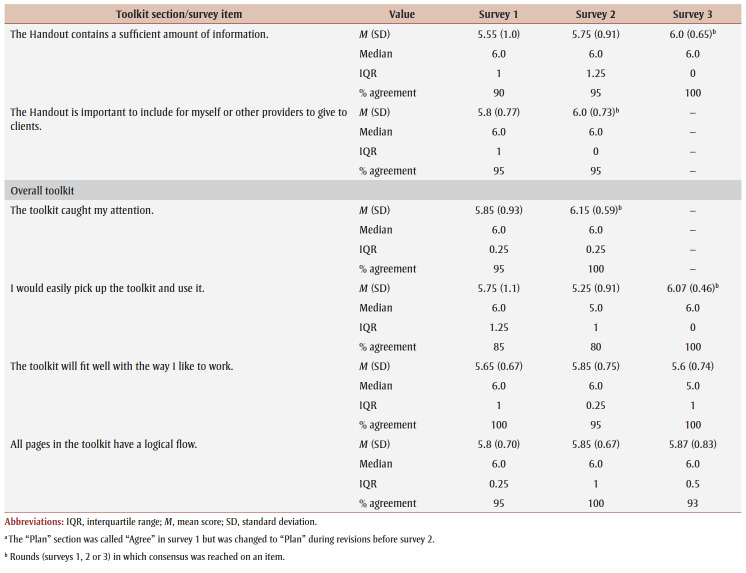

The toolkit was revised in response to each comment, or to multiple comments in cases where participants made similar statements. Specifically, we tried to improve the visual appeal (i.e. via bullet points, checkboxes and streamlined colours, graphics and fonts) and usability (i.e. by deleting redundant or unnecessary text, restructuring the “Counsel” section in the Tool and the User Guide, and clarifying wording, including by renaming the section “Agree” to “Plan”).

In survey 2, participants reached consensus on 17% (14/83) of the items and made 19 qualitative comments. How easy the title was to understand received the lowest mean score, 5.15 out of 7. Consensus was reached on the following 14 items: the visual appeal of the “Assess,” “Advise,” “Counsel” and “Arrange” sections, the User Guide and the Handout; the usability of the “Assess,” “Counsel” and “Arrange” sections; the “Arrange” section contained enough information; the User Guide was easy to understand; participants had sufficient knowledge to use the User Guide; it was important to include the Handout; and the overall toolkit caught PCPs’ attention. As a result, these 14 items were removed from survey 3. 

The participants’ qualitative comments indicated that the Preamble was not easy to understand and suggested rewording the Tool subtitle, tidying up the “Counsel” section, improving the visual appeal of the Tool and Handout, and providing more instructions in the “Plan” and “Counsel” sections (e.g. “May be helpful to specify that 1 is low confidence and 10 is high confidence” [P20]). Thus, the amount of text was further reduced, the graphics were simplified and enlarged, and the Tool subtitle and instructions for choosing a target behaviour, setting a goal and using the “Arrange” section were modified.

Consensus was reached on 55% (38/69) of the items in survey 3; however, the lowest mean score among the remaining items was 5.33 out of 7 (“The User Guide will make it easier to use the Whole Day Matters Tool”). No item received comments from more than one participant (i.e. <10% of participants), so subthreshold consensus was considered as having been reached for the remaining 45% of the items. Consensus was reached on the following 38 items: the Preamble contained enough information and improved the ability to use the toolkit; the visual appeal of the title and the “Ask” and “Plan” sections; the usability of the “Plan” section (for PCPs) and the Handout (for PCPs and adult clients); the title, “Ask,” “Advise” and “Plan” sections and the Handout made it easier to discuss movement behaviours; the “Assess,” “Advise” and “Arrange” sections and the Handout were easy to understand; PCPs would have sufficient knowledge to use the title, the “Ask,” “Assess,” “Advise,” “Plan” and “Counsel” sections and the Handout; PCPs would feel confident in their use of the “Assess,” “Advise” and “Arrange” sections, the User Guide and the Handout; the “Assess,” “Advise,” “Plan” and “Counsel” sections, the User Guide and the Handout contained enough information; it was important to include the “Advise” and “Counsel” sections; and the overall toolkit was easy to pick up and use.

Qualitative comments recommended rewording the toolkit title; restructuring the Preamble; emphasizing that the User Guide was for optional or temporary use; and making the Handout more accessible (based on comments such as the following: “For lower education patients or non-English speaking/English as a second-language patients, the Handout might be overwhelming or confusing” [P19]). The PCPs provided 12 qualitative comments, which were addressed by refining the wording in the “Arrange” section and restructuring the Preamble and Handout to improve readability.

Table 4 shows results of the concordance analyses. Results of the concordance analyses indicated a lack of concordance in survey 1 (*W* = 0.055, χ^2^ (82, 20) = 90.64, *p* = 0.241) and significant but poor levels of concordance in survey 2 (W = 0.099, χ^2^ (82, 20) = 162.50, *p*<0.001) and survey 3 (*W* = 0.177, χ^2^ (68, 15) = 180.60, *p*< 0.001). Poor levels of concordance may not necessarily indicate poor agreement or lack of consensus; rather, they indicate a larger range in participants’ responses. This increasing yet persistently poor concordance suggests that participants’ individual ratings still varied despite their increasing agreement at each survey.

**Table 4:** Associations between the Whole Day Matters Toolkit for Primary Care Providers consensus-building study participant ratings

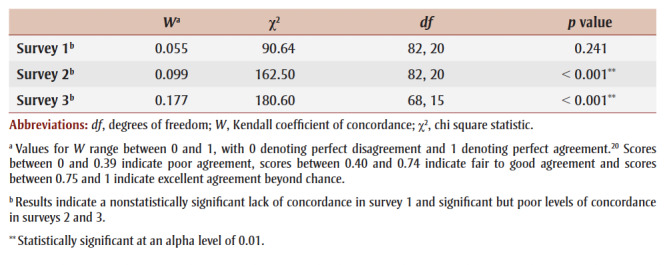

**Figure 2:**
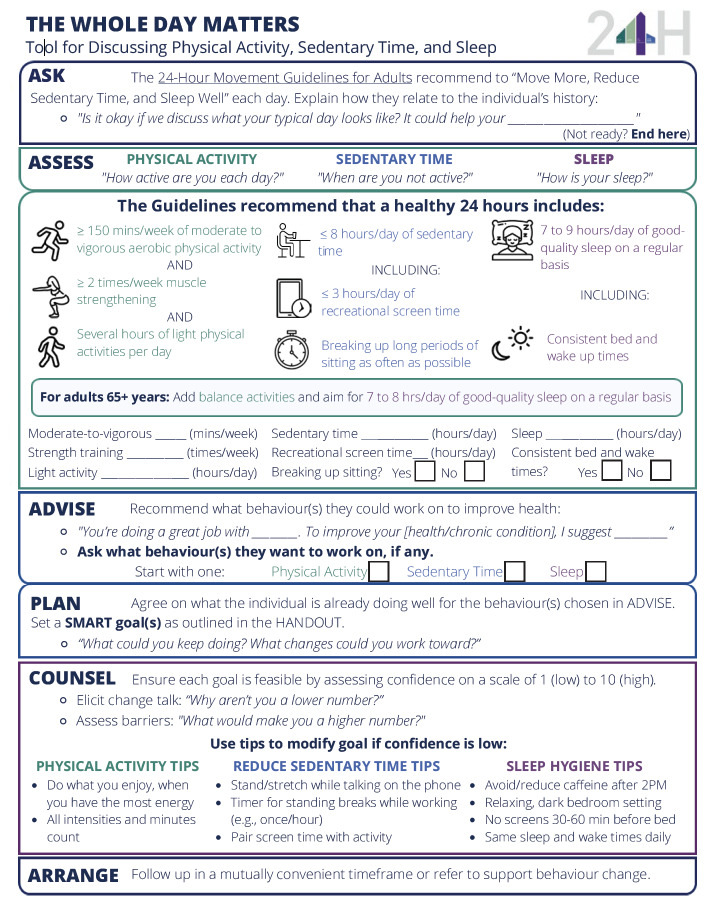
Sample page of the Whole Day Matters Toolkit for Primary Care Providers showing the one-page Tool

Prior to launch, the toolkit was translated into French and minor adjustments were made to improve accessibility, including increasing font size, adding alt text to images, ensuring logical reading order for screen reader use, and performing accessibility checks using Adobe Pro (Adobe Inc., San Jose, CA, US). On 21 September 2022, the toolkit was publicly launched on the CSEP website as a fillable PDF form available for free download (https://www.csepguidelines.ca/; see Figure 2 for a sample page).

***Dissemination***

One week post launch, the toolkit had been downloaded 493 times and the CSEP website had received 1291 unique page views (average time on page = 4:39 minutes, 2 minutes longer than other pages). Links to the toolkit across all dissemination channels were clicked 875 times. There were 8373 recipients of e-blasts and 13498 impressions (the number of times the 24HMG content was displayed across a user's feed, which includes multiple views from the same user), 405 Instagram and Facebook story views, 399 likes, 245 video views, 72 shares, 40 retweets and 15 comments across all social media platforms. At the 4-month follow-up (in January 2023), the toolkit had been downloaded 1072 times and there had been 2900 unique page views on the CSEP website (average time on page = 3:50 minutes). Over the 4 months since its release, the toolkit was the sixth most visited webpage on the CSEP guidelines website, surpassed only by the number of visits to the landing pages of the guidelines for the early years, children and youth, adults aged 18 to 64 years and adults aged 65 years and older.

## Discussion

The aim of this study was to gain consensus on the utility, acceptability and understandability (i.e. clarity) of the Whole Day Matters Toolkit for Primary Care, a resource aimed at mobilizing Canada’s 24HMGs in primary care practice, by adjusting parts of the toolkit as suggested by PCPs participating in this consensus-building study. Our findings support an increasing level of consensus among PCPs after three modified Delphi e-surveys. The percentage agreement criterion was met all of the time, whereas the mean score criterion was met 5%, 17% and 55% of the time in surveys 1, 2 and 3, respectively, signifying that this was the criterion that drove consensus. Consensus evolution was further supported by a decrease in qualitative comments, from 26 comments in survey1, to 19 in survey 2 and 12 in survey3. The qualitative comments in each survey guided toolkit revisions and helped ensure the working group was accurately interpreting participants’ quantitative ratings. We found subthreshold consensus on the remaining 45% of items in survey3, indicating that the majority of toolkit components were deemed acceptable among intended users. Indeed, the concordance analyses revealed poor concordance, demonstrating that participants’ opinions continued to differ despite increasingly supporting consensus. Collectively, these findings informed our toolkit dissemination efforts, where we highlighted how the toolkit could be applied differently based on PCPs’ unique roles and needs or the time available to them.

Research has highlighted the value of striving to understand dissent and using mixed methods in consensus-building approaches. In a recent paper, Shrier^26^ argued that interpreting both consensus and dissent can provide a more inclusive recognition of all participants’ opinions, as Delphi studies tend to inadvertently conceal dissenting opinions. In the present study, we strove to understand dissent and found it indicative of the various preferences of the participants, who came from nine different fields. Dissent may also represent a variety of well-documented barriers to movement behaviour promotion in primary care such as lack of time, competing priorities or limited training in movement behaviour promotion.^5^,^27^ For instance, participants in this study may not have reached consensus on the item “The ‘Counsel’ section will make it easier to discuss movement behaviours” because lack of time during clinical visits prevents movement behaviour counselling regardless of whether the “Counsel” section is usable.^5^ Moreover, Monforte et al.^28^ reported that Delphi methods may not capture sufficient depth of opinion from participants, and qualitative methods are required to provide a nuanced understanding. We attempted to add nuance by interpreting participants’ qualitative comments in parallel with their quantitative ratings, which guided toolkit revisions and helped convey why full consensus may not have been possible.

Tools have been previously created and used to help PCPs promote other public health guidelines. An example is the exercise prescription and referral tool available through the College of Family Physicians of Canada to promote Canada’s 2011 physical activity guidelines.^29^ However, consensus-building approaches have seldom been used prior to the implementation of public health promotion tools, limiting their utility and applicability in practice. Indeed, primary care discussions about health-promoting movement behaviours occur infrequently.^30^,^31^ In addition, most existing physical activity tools either omit sedentary behaviour or conflate it with physical inactivity. Overall, the integrated promotion of movement behaviours by health professionals continues to be a missed opportunity to broadly improve public health. Use of The Whole Day Matters toolkit could fill this gap by providing a flexible, usable, evidence-informed resource to support the dissemination and implementation of Canada’s 24HMGs in a wide range of health settings.

This study has several important practical and methodological implications. Practically, the toolkit is a resource that PCPs can use to more effectively promote the new Canadian public health guidelines and align their health promotion and preventive practices with a novel 24-hour approach.^7^

The present study advances tool development and consensus-building methodology, which can guide future tool development. Over 60% of participants at least “somewhat agreed” with all survey items, meaning this criterion did not impact consensus. Had we used a higher threshold (e.g. 70%), consensus would not have changed. The lowest percentage agreement score was 75%, which had a subthreshold mean score of 5.25 out of 7. Thus, this item would not have reached consensus anyway based on the mean score. Combining a higher percentage agreement threshold with a higher mean (e.g. 6 on a 7-point scale) would ensure that both a larger portion of participants “agree” and that participants who “disagree” are shifting toward “neither agree nor disagree” (i.e. “strongly disagree” less). Thus, in line with other researchers, we recommend using a 70% cut-point in future studies.^32^

***Strengths and limitations***

A primary strength of this study was our coproduction approach, which allowed us to consider multiple decision-maker perspectives and ensure that we were incorporating the varying preferences of PCPs.^12^ Another strength was the use of the modified 5As counselling framework to inform the development of the toolkit;^8^ this approach is often used by PCPs and further strengthens the toolkit’s utility in practice.^33^

This study also has limitations. Some professions who could work in primary care (e.g. kinesiologists, physiotherapists) were excluded. In Canada, there is no established mandate for the consistent, widespread integration of exercise professionals (e.g. kinesiologists, clinical exercise physiologists) or physiotherapists in family practices. Nevertheless, chronic disease prevention and management are central to the exercise physiology and physiotherapy professions in both public health and health care settings.^34^,^35^ For this reason, in the “Arrange” section of the Tool we identified exercise professionals to whom clients can be referred for follow-up movement behaviour support. We have also adapted the toolkit for physiotherapists in Canada since the present study (https://csepguidelines.ca/the-whole-day-matters-toolkit-for-physiotherapists/). Furthermore, while we attempted to recruit a national sample of PCPs, only PCPs working in British Columbia, Alberta and Ontario responded to the invitation to participate and were deemed eligible for the study. The sample also largely comprised PCPs who served urban communities and met the 24HMG recommendations of 150 minutes or more per week of moderate-to-vigorous physical activity. Indeed, challenges to social media recruitment strategies have been noted, including difficulties obtaining engagement and the need for existing social networks to be diverse and functional.^21^ Thus, the small sample size may have been a result of our use of social media to recruit PCPs and short recruitment window. Limitations of the low sample size include potential bias of results and reduced generalizability. Accordingly, the findings of this study may not capture the concerns of PCPs who engage in less physical activity and/or work in rural communities, other provinces or the territories in Canada; these PCPs may take different approaches to discussing the 24HMGs.

Notably, it was not possible to include all relevant information on movement behaviour promotion in the toolkit as the limited space prevented showing the extent of revisions (e.g. the “Arrange” section of the User Guide had to be restricted to four lines to keep the guide down to one page in length). Further, balancing varying views was challenging; some PCPs proposed removing content while others wanted more information on the same content. Finally, we did not evaluate toolkit implementation or its efficacy in increasing knowledge, skill, confidence and frequency of discussions about the 24HMGs in primary care.

Future research could explore what characteristics influence toolkit implementation by mapping PCPs’ barriers to using the toolkit and intervention strategies onto established behaviour change theories, models and frameworks (e.g. Capability, Opportunity, Motivation—Behaviour (COM-B) model, Behaviour Change Wheel).^36^ Chart reviews, questionnaires, recorded encounters and/or accelerometry could be conducted to assess how effective the toolkit is at enhancing PCPs’ knowledge, attitudes, confidence, skill and frequency of discussions about the 24HMGs and bringing about positive behaviour changes among adults in their care. The acceptability of the toolkit should continue to be assessed to identify what adaptations may be needed to ensure its continued utility among diverse PCPs.

## Conclusion

The 24HMGs are new public health guidelines in Canada that outline how adults can optimize their physical activity, sedentary behaviour and sleep to make their “whole day matter.” Strategic knowledge mobilization efforts ensure the 24HMGs implementation in PCPs’ practice, and future feasibility and effectiveness studies are needed to evaluate their public health impact. *The Whole Day Matters Toolkit for Primary Care* provides a suite of resources that a sample of PCPs in Canada have agreed can be used to enhance the health of the population. The toolkit is informed by evidence and behaviour change principles to support movement behaviour promotion. The whole toolkit or component pages can be used to help PCPs strengthen their health promotion services and improve public health.

## Acknowledgements

The authors would like to thank Dr. Jean-Philippe Chaput, Dr. Lora Giangregorio, Dr. Ian Janssen, Dr. Robert Ross and Dr. Travis Saunders for their input on verifying the accuracy of the content related to the 24HMGs in the final version of the Whole Day Matters Toolkit for Primary Care; Sophia Pourmatin for assisting with data analysis and tabulation; and Natara Ng Cheng Hin for piloting the surveys.

## Funding

This work was funded by the Public Health Agency of Canada (grant number 1920-HQ-000004) and supported by the Canadian Society for Exercise Physiology. Working group members received no financial compensation for their involvement in this study.

## Conflicts of interest

Taylor McFadden works for the Canadian Medical Association. However, the opinions and conclusions expressed are the writers’ own and not those of the Canadian Medical Association. 

There are no other conflicts of interest to declare.

## Authors’ contributions and statement

TLM: Conceptualization, methodology, validation, formal analysis, investigation, data curation, writing – original draft, writing – review and editing, visualization, project administration

MSF: Conceptualization, visualization, supervision, methodology, writing –review and editing

RJ: Conceptualization, visualization, methodology, writing – review and editing

KNL: Conceptualization, visualization, writing – review and editing

KM: Conceptualization, visualization, writing – review and editing

TM: Conceptualization, visualization, writing – review and editing, resources

JP: Conceptualization, visualization, methodology, writing – review and editing

JR: Visualization, writing – review and editing

ZJW: Visualization, writing – review and editing, resources

JRT: Conceptualization, visualization, supervision, methodology, writing – review and editing, funding acquisition

The content and views expressed in this article are those of the authors and do not necessarily reflect those of the Government of Canada.
